# Association of Matrix Metalloproteinase 1 and 3 (MMP1 and MMP3) gene polymorphisms with susceptibility to ESRD risk in North Indian population

**DOI:** 10.1186/1755-8166-7-S1-P30

**Published:** 2014-01-21

**Authors:** Archana Verma, Aneesh Shrivastava, Rama D Mittal

**Affiliations:** 1 Department of Urology and Renal Transplantation, SGPGIMS, Lucknow, India

## Background

End Stage Renal Disease (ESRD) is influenced by genetic and epigenetic factors. Few studies have been reported earlier depicting the role of Matrix Metalloproteinase in different populations for ESRD risk, but we have reported few novel and functional genes in North Indian population. The prime objective of the present study was to find out the association of four variants viz. MMP1-519(A/G) and MMP1-1607(1G/2G) and MMP3+5356(A/G) and MMP3 -1161(A/G) with ESRD risk.

## Materials and methods

4 SNPs were genotyped by Polymerase Chain Reaction-Restriction Fragment Length Polymorphism (PCR-RFLP) in 200 ESRD patients and 200 healthy controls, that were age and gender matched. To evaluate the SNP effects on ESRD, odds ratio (OR) and confidence interval (CI) 95% were calculated by SPSSver16.0. Haplotype analysis was done for MMP1 by SNPanalyzer1.2 ver.

## Results

In case of MMP1-519(A/G) the variant genotype GG showed significant reduced risk of ESRD (p=0.005, OR=0.254). On combining the heterozygous and variant genotypes marginal reduced risk was observed (p=0.055, OR=0.677), although we found significance reduced risk of ESRD (p=0.007, OR=0.639) in G allele. In case of MMP1-1607(1G/2G) the variant genotype 2G2G showed significant reduced risk of ESRD (p=0.008, OR=0.476) whereas the combination of heterozygous and variant genotypes reduced risk was obtained (p=0.027, OR=0.614). At allelic level, 2G allele showed reduced risk (p=0.006, OR=0.675) of ESRD. Gene-gene interaction was also performed for above gene variants. We found a significant reduced risk in combination GG+AA (p=0.034, OR=0.185) on interacting MMP1-519(A/G) and MMP3-1161(A/G). We also found reduced risk for ESRD for MMP1-519(A/G) and MMP1-1607(1G/2G) while looking risk for ESRD at haplotype level. Bioinformatics analysis (in-silico) showed no association with ESRD.

## Conclusions

Our results indicated that polymorphism in MMP1-519(A/G) and MMP1-1607(1G/2G) showed reduced risk for ESRD in North-Indian population. More relevant studies with large sample size and diverse ethnicity are required to validate these observations.

